# Cardiac myosin regulatory light chain kinase modulates cardiac contractility by phosphorylating both myosin regulatory light chain and troponin I

**DOI:** 10.1074/jbc.RA119.011945

**Published:** 2020-02-21

**Authors:** Ivanka R. Sevrieva, Birgit Brandmeier, Saraswathi Ponnam, Mathias Gautel, Malcolm Irving, Kenneth S. Campbell, Yin-Biao Sun, Thomas Kampourakis

**Affiliations:** ‡ Randall Centre for Cell and Molecular Biophysics and British Heart Foundation Centre of Research Excellence, King's College London, London SE1 1UL, United Kingdom; § Department of Physiology, College of Medicine, University of Kentucky, Lexington, Kentucky 40536–0298

**Keywords:** cardiomyocyte, posttranslational modification (PTM), myosin, troponin, contractile protein, calcium signalling, cardiac muscle regulation, cardiac myosin light chain kinase, heart function, troponin I phosphorylation

## Abstract

Heart muscle contractility and performance are controlled by posttranslational modifications of sarcomeric proteins. Although myosin regulatory light chain (RLC) phosphorylation has been studied extensively *in vitro* and *in vivo*, the precise role of cardiac myosin light chain kinase (cMLCK), the primary kinase acting upon RLC, in the regulation of cardiomyocyte contractility remains poorly understood. In this study, using recombinantly expressed and purified proteins, various analytical methods, *in vitro* and *in situ* kinase assays, and mechanical measurements in isolated ventricular trabeculae, we demonstrate that human cMLCK is not a dedicated kinase for RLC but can phosphorylate other sarcomeric proteins with well-characterized regulatory functions. We show that cMLCK specifically monophosphorylates Ser^23^ of human cardiac troponin I (cTnI) in isolation and in the trimeric troponin complex *in vitro* and *in situ* in the native environment of the muscle myofilament lattice. Moreover, we observed that human cMLCK phosphorylates rodent cTnI to a much smaller extent *in vitro* and *in situ*, suggesting species-specific adaptation of cMLCK. Although cMLCK treatment of ventricular trabeculae exchanged with rat or human troponin increased their cross-bridge kinetics, the increase in sensitivity of myofilaments to calcium was significantly blunted by human TnI, suggesting that human cTnI phosphorylation by cMLCK modifies the functional consequences of RLC phosphorylation. We propose that cMLCK-mediated phosphorylation of TnI is functionally significant and represents a critical signaling pathway that coordinates the regulatory states of thick and thin filaments in both physiological and potentially pathophysiological conditions of the heart.

## Introduction

Cardiac muscle contraction is driven by the cyclic interactions of myosin and actin coupled to ATP hydrolysis. Calcium binding to troponin triggers activation of the actin-containing thin filament mediated by the azimuthal movement of tropomyosin on its surface, which allows myosin head domains from the neighboring myosin-containing thick filaments to strongly attach to available actin-binding sites. Subsequently, small conformational changes in the catalytic part of the myosin head domain, associated with the release of P_i_, are amplified by the myosin light chain–containing “lever arm” or light chain domain, which results in nanometer-scale displacement of the thin filaments toward the center of the sarcomere or generation of piconewton-scale forces (1).

Heart muscle contractility and performance are also controlled by posttranslational modifications of sarcomeric proteins, including phosphorylation of the myosin regulatory light chain (RLC)^2^ by the cardiac isoform of myosin light chain kinase (cMLCK). RLC phosphorylation has been shown to be an important regulator of cardiac muscle function, with ablation of cMLCK or phosphorylation of RLC *per se* leading to cardiac muscle dysfunction and pathological hypertrophy in animal models (2–4). In contrast, increasing RLC phosphorylation in transgenic animal models has a cardioprotective function (5). RLC phosphorylation by cMLCK increases the calcium sensitivity, isometric force, and cross-bridge kinetics of isolated cardiac muscle fibers (6, 7) and has been proposed to control cardiac twitch dynamics and inotropy in the intact heart (8). Moreover, RLC phosphorylation has been shown to modify cardiac muscle length-dependent activation, the cellular analog of the Frank–Starling mechanism (7, 9).

cMLCK is a member of the Ca^2+^ and calmodulin-dependent protein kinase family; it has a C-terminal canonical calmodulin-binding site and Ca^2+^/calmodulin-dependent activity (10, 11). The conserved catalytic domain has high similarity to that of smooth and skeletal muscle MLCK. However, cMLCK exhibits a unique N-terminal region with so far unknown structure and function, although sequence analysis revealed the presence of several putative phosphorylation sites for kinases known to regulate myofilament function, such as PKA and PKC (12). In the vertebrate heart, cMLCK is the principal kinase acting on RLC, and no other substrates have been identified to date, which led to the suggestion that cMLCK is a dedicated kinase with specific cellular functions (13). Moreover, overexpression of cMLCK in isolated cardiomyocytes increases sarcomere organization, whereas its knockdown results in sarcomere disassembly via so far uncharacterized mechanisms (11, 14).

The clinical significance of cMLCK is highlighted by the fact that mutations in the gene encoding for cMLCK (*MLYK3*) have been associated with development of dilated cardiomyopathy in humans (15, 16), and increased protein turnover of cMLCK has been suggested to underlie the transition from compensatory hypertrophy to decompensated heart failure in animal models (17). In contrast, increasing RLC phosphorylation prevents development of hypertrophic cardiomyopathy-associated heart failure (18) and increases cardiac output, suggesting that cMLCK may prevent clinical onset of cardiomyopathies and increase the performance of the failing heart. However, the exact role of cMLCK in cardiomyocyte contractile regulation and sarcomere organization remains to be established.

In this study, we show that cMLCK is not a dedicated kinase and, in fact, phosphorylates human cardiac troponin I, an important regulator of myofilament calcium sensitivity and relaxation kinetics. We show that cMLCK specifically monophosphorylates human cTnI on serine 23 *in vitro* in isolated protein preparations and *in situ* in the native environment of the muscle lattice. Moreover, cTnI phosphorylation by cMLCK is species-specific, and cMLCK does not phosphorylate cTnI in rodent muscle because of sequence variations around the phosphorylatable serine residue. We also show that the functional consequences of cMLCK activation differ significantly in the presence of either human or rodent troponin, which, in the case of the former, modifies the functional effects of RLC phosphorylation observed previously in isolated cardiac fibers from rodent hearts (7). Our results provide new mechanistic insights into the regulation of cardiac muscle contractility by cMLCK and its species-specific differences.

## Results

### cMLCK phosphorylates serine 23 of human cTnI in vitro

**Figure 1.**
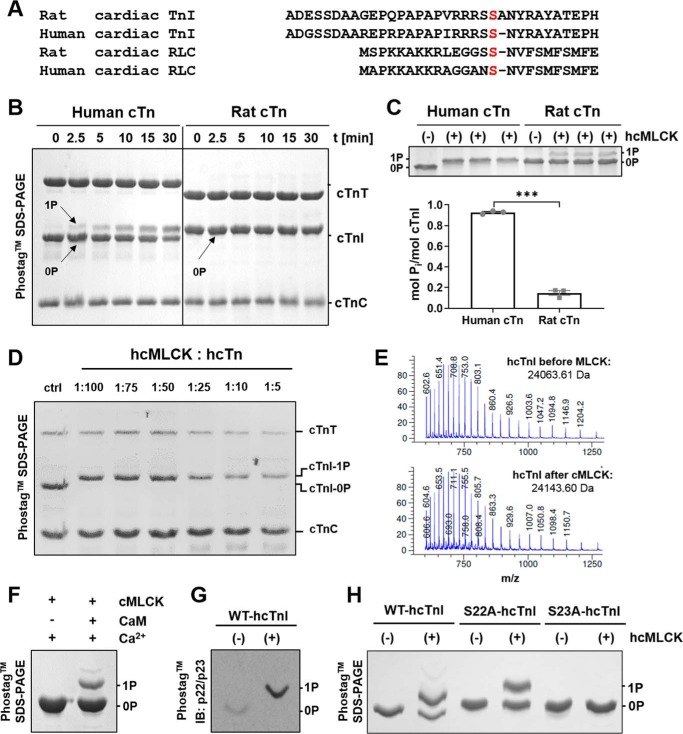
***In vitro* phosphorylation of human cardiac troponin I on serine 23 by cMLCK.** *A*, protein primary sequence alignment of the N-terminal extension of rat and human cTnI with the sequence surrounding the phosphorylated serines in rat and human cRLC. Phosphorylatable serines are highlighted in *red. B*, *in vitro* kinase assays of human (*left*) and rat cardiac troponin (*right*), with cMLCK analyzed by Phos-tag^TM^ SDS-PAGE. Troponin T, I, and C are labeled accordingly. *C*, densitometric analysis of human and rat cTnI phosphorylation levels after more than 60 min of incubation of troponin complexes with cMLCK. *D*, kinase assays at different human cTn to cMLCK stoichiometries analyzed by Phos-tag SDS-PAGE. cMLCK phosphorylated cTnI to ∼1 mol P_i_/mol cTnI under all conditions tested. *ctrl*, control. *E*, ESI-MS analysis of cTnI before (*top*) and after incubation of cTn complex with cMLCK (*bottom*, 1:5 enzyme:substrate ratio) from *D*. The increase in mass by ∼80 Da indicates monophosphorylation. *F* and *G*, *in vitro* kinase assay of isolated hcTnI with cMLCK (*F*) followed by Western blotting (*G*) using a pSer^22^/pSer^23^-specific antibody. *H*, alanine substitution of serine 22 or 23 demonstrates that cMLCK specifically phosphorylates serine 23 in the hcTnI N-terminal extension. Means ± S.E. (*n* = 3). Statistical significance of difference was assessed with an unpaired, two-tailed Student's *t* test: ***, *p* < 0.001.

We compared the primary sequence of known myofilament phosphoproteins from different species with the cMLCK consensus sequence in RLC and identified serines 22/23 in the cardiac specific N-terminal extension (NTE) of human cardiac troponin I (hcTnI) as potential substrates (Fig. 1*A*, *red*). In contrast, rodent cardiac troponin I differs considerably in its primary sequence surrounding the phosphorylatable serines 22/23 in its NTE, suggesting that rodent (including rat and mouse) cTnI might not be a substrate for cMLCK. The phosphorylatable serines 22/23 are followed by an asparagine residue (P+1 position) in human cTnI (Fig. 1*A*), which is similar to the consensus sequence found in rodent and human cardiac RLC. In contrast, in rodent cTnI, the phosphorylatable serines are followed by an alanine in the P+1 and an asparagine in the P+2 position (Fig. 1*A*), which might interfere with substrate recognition by cMLCK.

To test this hypothesis, we used recombinant human or rat cardiac troponin complex as substrates in cMLCK *in vitro* kinase assays and analyzed their phosphorylation profiles by Phos-tag^TM^ SDS-PAGE. As shown in Fig. 1*B*, cMLCK specifically phosphorylated hcTnI to ∼0.6 mol P_i_/mol cTnI within 30 min of incubation. No phosphorylation of cardiac troponin T or C was observed under the same conditions, suggesting that cMLCK has a high specificity toward hcTnI *in vitro*. In contrast, rat cardiac troponin I (rcTnI) was phosphorylated to less than 0.02 mol P_i_/mol cTnI under the same conditions, suggesting that it is a poor substrate for cMLCK *in vitro*. Note that unphosphorylated rat cTnT and cTnI migrate faster and slower in SDS-PAGE, respectively, than the corresponding human proteins. Prolonged incubation of both troponin complexes for more than 60 min resulted in almost complete monophosphorylation of hcTnI (>0.9 mol P_i_/mol cTnI) but less than 0.15 mol P_i_/mol cTnI phosphate incorporation for the rat troponin complex (Fig. 1*C*). In contrast to cTnI, cMLCK monophosphorylates isolated human and rat cRLC under the same conditions, with a similar time course to ∼1 mol P_i_/mol cRLC within 60 min (10).

Next we tested the specificity of cMLCK for hcTnI by incubating human cardiac troponin complex with increasing concentrations of cMLCK (*i.e.* from 1:100 to 1:5 enzyme:substrate ratio) for 1 h and analyzed the phosphorylation profiles of cardiac troponin T, C, and I by Phos-tag^TM^ SDS-PAGE and electron spray ionization (ESI) MS (Fig. 1, *D* and *E*, and Table S1). Only hcTnI was specifically monophosphorylated to ∼1 mol P_i_/mol cTnI under all conditions tested, supporting the hypothesis that cMLCK specifically phosphorylates a single residue in cTnI, *i.e.* either serine 22 or 23. We confirmed this result by phosphorylating isolated hcTnI with cMLCK (Fig. 1*F*), followed by Phos-tag^TM^ SDS-PAGE and Western blotting using a cTnI pSer^22^/pSer^23^-specific antibody (Fig. 1*G*). The phosphospecific antibody clearly recognized the monophosphorylated species, supporting the above conclusion that Ser^22^, Ser^23^, or both are substrates for cMLCK *in vitro*. To identify the phosphorylated serine residue, we incubated isolated WT hcTnI or hcTnI with Ser^22^ or Ser^23^ substituted by alanine with Ca^2+^/calmodulin-activated cMLCK (Fig. 1*H*). Only WT and S22A-substituted cTnI were phosphorylated by cMLCK, localizing the phosphorylation site to Ser^23^, in good agreement with the sequence similarity to RLC (Fig. 1*A*). A minor bis-phosphorylated human cardiac troponin I species was observed when the protein was used in isolation in kinase assays (Fig. 1, *F* and *H*), suggesting that cMLCK phosphorylated a secondary unspecific site that is, however, not accessible in the trimeric troponin complex.

A sequence alignment of the N-terminal extension of cTnI from different species revealed that the P+1 position is an asparagine in all Old and New World primate species and humans (Fig. S2) and an alanine in other vertebrates, suggesting that cTnI phosphorylation by cMLCK might be a specific evolutionary adaptation.

### In situ phosphorylation of cTnI by cMLK

**Figure 2.**
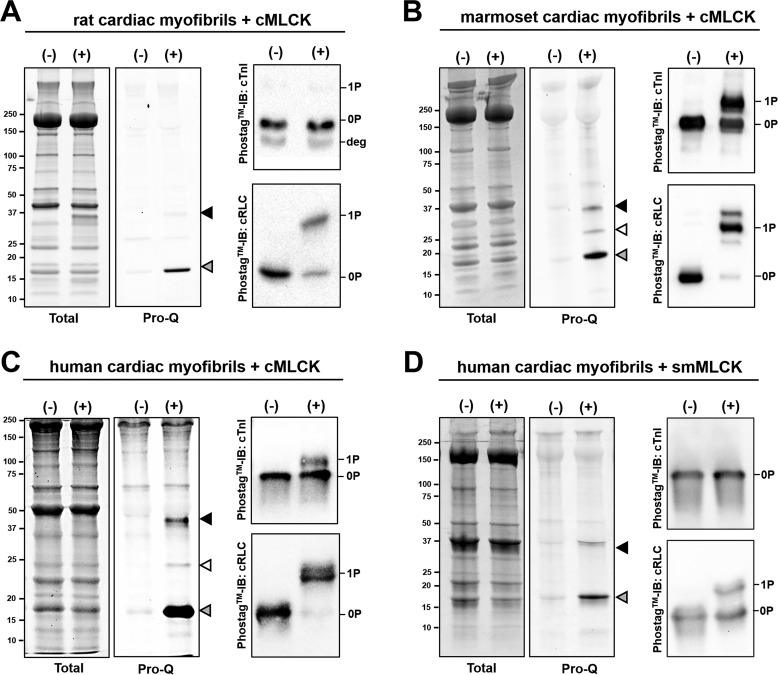
***In situ* phosphorylation of cTnI and cRLC by cMLCK in native cardiac myofibrils.** *A*, Pro-Q diamond and total protein staining (*left*) of λ protein phosphatase–treated rat cardiac myofibrils before (−) and after cMLCK incubation (+). The *black* and *gray arrowheads* indicate phosphorylated cMLCK and cRLC, respectively. Cardiac cTnI and cRLC phosphorylation levels were further confirmed by Phos-tag^TM^ SDS-PAGE and Western blotting (*right*). Degradation products of cTnI are labeled accordingly (*deg*). *IB*, immunoblot. *B*, Pro-Q diamond and total protein staining (*left*) of λ protein phosphatase–treated marmoset CMFs before (−) and after cMLCK incubation (+). The *black*, *white*, and *gray arrowheads* indicate phosphorylated cMLCK, cTnI, and cRLC, respectively. Cardiac cTnI and cRLC phosphorylation levels in the same samples were further confirmed by Phos-tag^TM^ SDS-PAGE and Western blotting (*right*). *C* and *D*, the same assays as in *B* but using cardiac myofibrils isolated from human ventricular tissue treated with either cardiac (*C*) or smooth muscle MLCK (*D*).

Next we confirmed the results for the isolated proteins described above in experiments with isolated cardiac myofibrils (CMFs), in which intact thin and thick filaments are organized into the native myofilament lattice. CMFs isolated from rat ventricular tissue were pretreated with λ protein phosphatase (λPP) to homogenously reduce protein phosphorylation levels. λPP treatment resulted in efficient dephosphorylation of cRLC, cTnI, cardiac troponin T, desmin, and cMyBP-C, as assessed by SDS-PAGE and Pro-Q Diamond phospho-protein staining (Fig. S3). Myofibrillar proteins after λPP treatment or sequential treatment with λPP followed by incubation with Ca^2+^/CaM/cMLCK for 1 h were separated by SDS-PAGE, and phosphorylated proteins were identified by phospho-protein staining with Pro-Q Diamond and total protein staining with Coomassie or SYPRO Ruby. As described previously (7), cMLCK specifically phosphorylated an abundant rat myofilament protein with an apparent molecular mass of ∼18 kDa (Fig. 2*A*, *gray arrowhead*), in good agreement with the calculated mass of cRLC. An additional band in the Pro-Q staining with an apparent molecular mass of ∼37 kDa (Fig. 2*A*, *black arrowhead*) corresponds to cMLCK, which is endogenously phosphorylated (10). We confirmed cRLC phosphorylation in treated rat CMFs by Phos-tag^TM^ SDS-PAGE and Western blot against cRLC (Fig. 2*A*, *right*). As expected, cRLC was highly phosphorylated after cMLCK treatment (∼0.7 mol P_i_/mol cRLC). In contrast, Phos-tag^TM^ Western blotting against cTnI with the same samples showed that, although λPP treatment resulted in efficient dephosphorylation of cTnI, the rat variant was not phosphorylated by cMLCK *in situ*.

We repeated the *in situ* phosphorylation experiments in CMFs prepared from marmosets (*Callithrix jacchus*) and human left ventricles, which exhibit a significantly higher cTnI primary sequence identity (∼92% rat *versus* human cTnI and ∼96% marmoset *versus* human cTnI; Fig. S2), including an asparagine in the P+1 position following serine 23 in the NTE. Pro-Q phosphoprotein staining of λPP/cMLCK-treated marmoset and human CMFs showed three bands with apparent molecular masses of ∼18, ∼24. and ∼37 kDa (Fig. 2, *B* and *C*, *gray*, *white*, and *black arrowheads*, respectively), consistent with the expected molecular masses of cRLC, cTnI, and cMLCK, respectively. As before, we used Phos-tag^TM^ SDS-PAGE and Western blotting to confirm that cRLCs in both sets of CMFs were phosphorylated after cMLCK treatment (∼0.8 mol P_i_/mol cRLC). However, in contrast to rat CMF, cTnI was phosphorylated in the same samples to about 0.3–0.6 mol P_i_/mol cTnI (Fig. 2*B*, *right*), confirming that cMLCK phosphorylates native marmoset and human cTnI in the myofilament lattice *in situ*. Specific phosphorylation of marmoset and human cTnI at serine 23 *in situ* was further confirmed by Western blotting using a pSer^22^/pSer^23^-specific antibody (Fig. S4*A*). Incubation of human CMFs with smooth muscle MLCK did not result in phosphorylation of cTnI, suggesting that phosphorylation of cTnI is specific to the cardiac isoform of MLCK (Fig. 2*D*).

**Figure 3.**
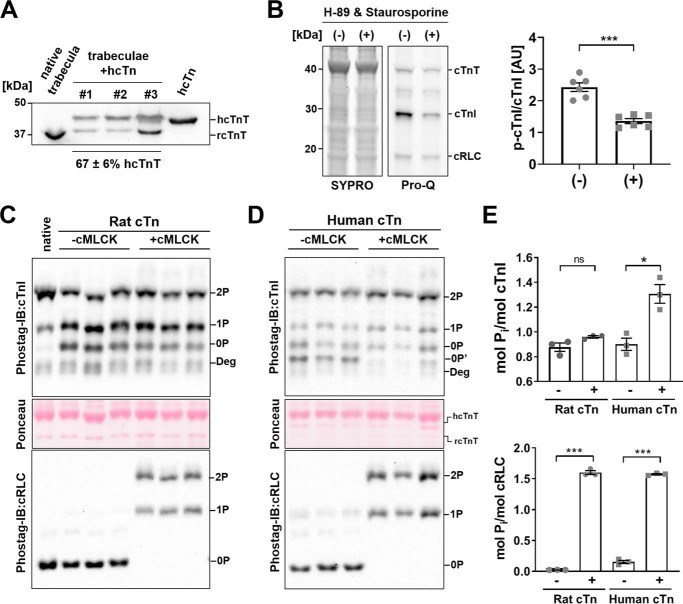
***In situ* phosphorylation of cTnI and cRLC by cMLCK in troponin-exchanged rat ventricular trabeculae.** *A*, recombinant cardiac troponin exchange efficiency in ventricular trabeculae, determined by Western blotting against cTnT. Endogenous native rat cTnT (*left*) migrates faster in SDS-PAGE than recombinant human cTnT (*right*), allowing estimation of the cTn exchange efficiency into rat ventricular trabeculae (*center*). Mean ± S.E., *n* = 3. *B*, low-molecular-weight portion of SDS-PAGE of ventricular trabeculae treated without (−) and with (+) protein kinase inhibitors (H-89 and staurosporine). Gels were stained with phospho-specific Pro-Q Diamond or SYPRO total protein stain. Densitometric analysis for cTnI is shown on the *right*. Means ± S.E., *n* = 6. *C* and *D*, rat cTn-exchanged (*C*) or human cTn-exchanged (*D*) demembranated ventricular trabeculae were incubated without (−*cMLCK*, time-matched control) or with cMLCK (+*cMLCK*), and phosphorylation levels were determined by Phos-tag^TM^ SDS-PAGE, followed by Western blotting against cTnI (*top*) and cRLC (*bottom*). Unphosphorylated human cTnI (indicated by *0P'*) migrates faster than unphosphorylated rat cTnI (*0P*). TnI degradation products are labeled accordingly (*Deg*). Ponceau stains for endogenous rat (*rcTnT*) and exogenous human cardiac troponin T (*hcTnT*) are shown in the *center panels. E*, densitometric analysis of cTnI (*top*) and cRLC phosphorylation (*bottom*) levels before (−) and after cMLCK (+) incubation in *C* and *D*. Means ± S.E., *n* = 3–6. Statistical significance of differences was assessed with an unpaired, two-tailed Student's *t* test. *ns*, not significant; *, *p* < 0.05; ***, *p* < 0.001.

To assess the functional consequences of hcTnI phosphorylation by cMLCK, we exchanged unphosphorylated rat or human cardiac troponin complex into demembranated rat right ventricular trabeculae with a roughly 70% exchange efficiency (Fig. 3*A*). Endogenous cTnI in demembranated ventricular trabeculae is highly phosphorylated because of residual kinase activity in the quiescent preparations (Fig. 3*B*), and overnight exchange with the unphosphorylated recombinant troponins reduced cTnI phosphorylation from ∼1.8 mol P_i_/mol cTnI to ∼0.9 mol P_i_/mol cTnI in both groups (Fig. 3, *C* and *D*). As described previously, the level of RLC phosphorylation was low in demembranated trabeculae (<0.05 mol P_i_/mol RLC) (10), and recombinant troponin exchange had no further effect (Fig. 3, *C* and *D*, *bottom*). Incubation in activating solution (*p*Ca 4.5) containing 25 μmol/liter blebbistatin and 1 μmol/liter cMLCK/CaM had no effect on the cTnI phosphorylation level of rat troponin-exchanged trabeculae but significantly increased cTnI phosphorylation in the presence of human cardiac troponin to ∼1.3 mol P_i_/mol cTnI (Fig. 3, *D* and *E*, *top*). In contrast, RLC was highly phosphorylated in both groups of trabeculae after cMLCK treatment (Fig. 3, *D* and *E*, *bottom*). In summary, these results show that cMLCK phosphorylates hcTnI *in situ* in the native environment of the myofilament lattice.

### Functional consequences of cTnI phosphorylation by cMLCK in cardiac muscle

The functional consequences of hcTnI phosphorylation by cMLCK were assessed by measuring the calcium sensitivity and cross-bridge kinetics of troponin-exchanged ventricular trabeculae before and after incubation with cMLCK/Ca^2+^/CaM. However, exchange of ∼70% endogenous with recombinant troponin had only a moderate effect on the cTnI phosphorylation level observed after exchange, with about 30% still present as the monophosphorylated and 30% as the bis-phosphorylated form (Fig. 3, *C* and *D*), suggesting that recombinant unphosphorylated cTnI was partially phosphorylated during the protein exchange by kinases still present in the freshly prepared ventricular trabeculae (Fig. 3*B*). Because of the high basal level of phosphorylation, the effects of kinase treatment on cTnI phosphorylation and myofilament function could not be unambiguously interpreted. We therefore employed an additional dephosphorylation step, using λPP as described previously for mouse ventricular trabeculae (19).

**Figure 4.**
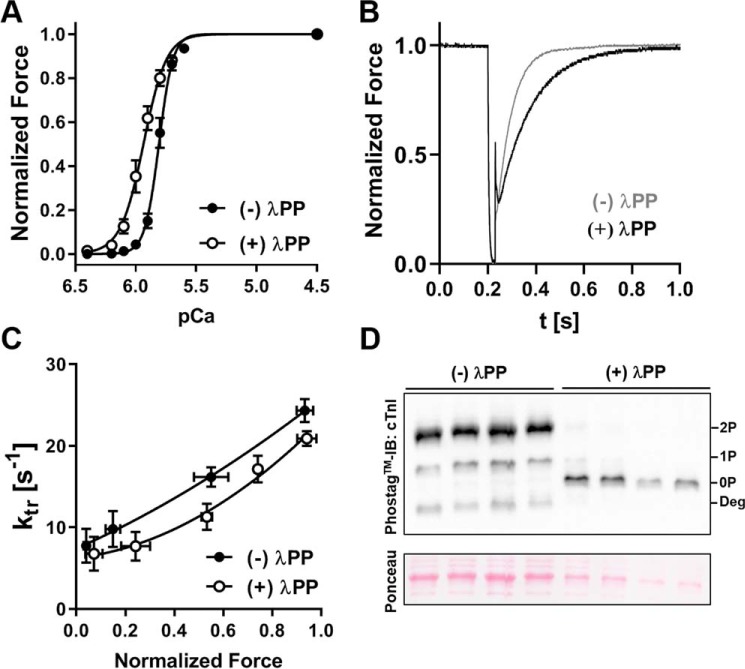
**Effect of λPP treatment on cardiac muscle mechanics.** *A*, force–*p*Ca relation of demembranated rat ventricular trabeculae before (*closed circles*) and after λPP treatment (*open circles*). *B*, representative recordings of force redevelopment after the release–restretch protocol before (*gray*) and after λPP treatment (*black*) at ∼50% maximal activation. *C*, force–*k*_tr_ relation of demembranated rat ventricular trabeculae before (*closed circles*) and after λPP treatment (*open circles*). *D*, *top*, cTnI phosphorylation levels of control and experimental trabeculae that underwent λPP treatment analyzed by Phos-tag^TM^ SDS-PAGE and Western blotting against cTnI. *Deg*, cTnI degradation products. *Bottom*, Ponceau stain showing the actin loading control for individual trabeculae. Means ± S.E., *n* = 3–4.

First we tested the effects of λPP treatment on cardiac muscle mechanics. Incubation of rat ventricular trabeculae with λPP increased the calcium sensitivity of force, as indexed by an increase in *p*Ca_50_ from 5.81 ± 0.01 to 5.94 ± 0.01 (means ± S.E., *n* = 4), but decreased the steepness of the force–calcium relation (*n_H_* of 7.47 ± 0.56 *versus* 4.56 ± 0.37, mean ± S.E., *n* = 4) (Fig. 4*A*). Moreover, λPP treatment decreased the rate of force redevelopment at intermediate and high levels of activation (Fig. 4, *B* and *C*) but had little or no effect on passive or maximal calcium-activated isometric force. After the experiments, trabeculae were dismounted and dissolved in SDS-PAGE loading buffer, and cTnI phosphorylation levels were determined by Phos-tag^TM^ SDS-PAGE followed by Western blotting against cTnI (Fig. 4*D*). Trabeculae from the same hearts that did not undergo λPP treatment were used as negative controls. As before, endogenous cTnI phosphorylation levels in untreated control trabeculae were high (∼1.8 mol P_i_/mol cTnI), and incubation with λPP efficiently reduced cTnI phosphorylation to less than 0.1 mol P_i_/mol cTnI, in good agreement with the CMF experiments described above. λPP treatment also resulted in efficient dephosphorylation of cMyBP-C, which was confirmed by Western blotting using a phospho-specific antibody (Fig. S4*B*). Comparison of the protein phosphorylation levels with the functional effects suggests that λPP treatment increases calcium sensitivity and decreases cross-bridge kinetics of ventricular trabeculae via dephosphorylation of cTnI and cMyBP-C, respectively. Therefore, to achieve a homogeneously low cTnI phosphorylation level, we first exchanged trabeculae overnight with unphosphorylated human or rat cardiac troponin, followed by λPP treatment directly before the mechanical experiments and subsequent incubation with Ca^2+^/CaM/cMLCK.

**Figure 5.**
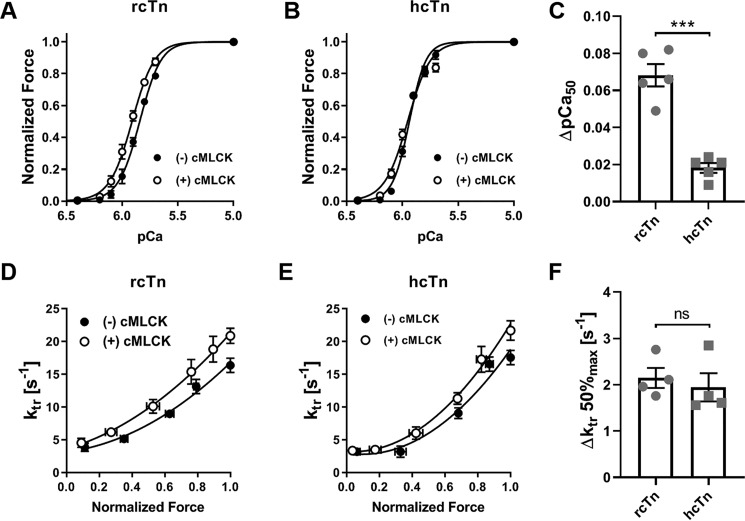
**Effect of cMLCK incubation on mechanical properties of rat and human cTn-exchanged rat ventricular trabeculae.** *A* and *B*, normalized force–*p*Ca relations of λPP-treated rat cTn-exchanged (*A*) and human cTn-exchanged (*B*) trabeculae before (*closed circles*) and after cMLCK incubation (*open circles*). *C*, bar graph indicating the increase in calcium sensitivity (Δ*pCa_50_*) upon cMLCK treatment in the two groups. *D* and *E*, force–*k*_tr_ relation of rat cTn-exchanged (*D*) and human cTn-exchanged (*E*) trabeculae before (*closed circles*) and after cMLCK incubation (*open circles*). *F*, bar graph indicating the increase in the rate of force redevelopment at 50% maximal activation (Δ*k_kr_ 50%_max_*) upon cMLCK treatment in the two groups. Means ± S.E., *n* = 4–5. Statistical significance of differences between the two groups was assessed with an unpaired, two-tailed Student's *t* test. *ns*, not significant; ***, *p* < 0.001.

Incubation of rat cTn-exchanged and λPP-dephosphorylated trabeculae with Ca^2+^/CaM-activated cMLCK increased the calcium sensitivity of force by 0.07 ± 0.01 *p*Ca (mean ± S.E., *n* = 5) (Fig. 5*A*, Tables S1 and S2) but had no effect on the steepness of the force calcium relation (*n_H_* of 4.76 ± 0.46 *versus* 4.44 ± 0.42, mean ± S.E.), in good agreement with results obtained previously from native rat ventricular trabeculae (7). This result suggests that cTn exchange and dephosphorylation with λPP did not affect the physiological response of trabeculae to RLC phosphorylation by cMLCK. In contrast, the increase in calcium sensitivity after cMLCK treatment was strongly reduced in trabeculae exchanged with human cardiac troponin to 0.02 ± 0.01 *p*Ca (mean ± S.E., *n* = 5) (Fig. 5, *B* and *C*, and Table S1), suggesting that hcTnI phosphorylation by cMLCK in the same preparations mitigated the increase in calcium sensitivity associated with phosphorylation of cRLC. Moreover, cMLCK treatment reduced the steepness of the force calcium relation from *n_H_* 5.51 ± 0.19 to 4.11 ± 0.25 (means ± S.E., *n* = 5) in the presence of human cTn. cMLCK treatment slightly increased passive and active force of human and rat cTn-exchanged trabeculae.

We determined the effect of cMLCK phosphorylation on cross-bridge kinetics by measuring the rate of force redevelopment (*k*_tr_) at different levels of activation by a rapid shortening–restretch maneuver. Rat and human cTn-exchanged trabeculae exhibited activation-dependent changes in *k*_tr_ (Fig. 5, *D* and *E*), ranging from ∼5 s^−1^ at low levels of activation to ∼15 s^−1^ at maximal activation. Data points were fitted to a quadratic equation to extract *k*_tr_ at half-maximal levels of force (*k*_tr_ 50%_max_). In human and rat cTn-exchanged trabeculae, cMLCK treatment increased *k*_tr_ 50%_max_ by ∼2 s^−1^ (Fig. 4*F* and Table S1), suggesting increased cross-bridge kinetics at intermediate and high levels of activation.

After the experiments, trabeculae were dismounted, and phosphorylation levels were determined by Phos-tag^TM^ SDS-PAGE and Western blotting (Fig. S4*C*). RLC was phosphorylated to 0.28 ± 0.08 mol P_i_/mol RLC (mean ± S.E., *n* = 5) and 0.36 ± 0.05 mol P_i_/mol cRLC in rat and human cTn-exchanged trabeculae, respectively. cTnI was largely dephosphorylated in rat cTn-exchanged trabeculae. Because monophosphorylated hcTnI and unphosphorylated rcTnI comigrate in Phos-tag^TM^ SDS-PAGE (see also Fig. 1*B*), we could not directly determine the level of troponin I phosphorylation, but it was estimated to be ∼0.3 mol P_i_/mol, assuming a troponin exchange efficiency of 70%.

## Discussion

### Cardiac myosin light chain kinase is not a dedicated kinase

Although myosin light chain kinases have long been considered “dedicated kinases” that specifically phosphorylate the myosin heavy chain-associated RLCs (13), recent research has suggested that MLCKs can phosphorylate other targets; *e.g.* the skeletal isoform of MLCK has been shown to regulate skeletal muscle myogenesis via phosphorylation of the transcription factor MEF2c (20), and smooth muscle MLCK has been suggested recently to phosphorylate a multitude of proteins that, interestingly, overlap with PKA signaling targets in nonmuscle cells (21).

In this study, we show that the human cardiac isoform of MLCK specifically monophosphorylates human cardiac troponin I in a Ca^2+^/calmodulin-dependent manner, in agreement with its Ca^2+^-dependent activity reported previously (10) (Fig. 1*F*). cMLCK phosphorylated cTnI on serine 23 not only on the isolated cTnI subunit but also when it was incorporated within the trimeric troponin complex or the myofilament lattice (Figs. 1–3), suggesting that cMLCK has a high specificity toward hcTnI serine 23.

The NTE of hcTnI (amino acids 1–30) displays a sequence motif (Fig. 1*A*) similar to that found in cMLCK's primary substrate RLC. Serine 23 in hcTnI is followed by an asparagine and an amphipathic amino acid residue (pSNY), which is similar to the conserved amino acid sequence found in the NTE of all RLC isoforms (pSNVF). Stepwise deletion of this conserved sequence in smooth muscle RLC decreased the maximal rate of phosphorylation, and it has been suggested that these residues contribute to substrate recognition by binding to a pocket found next to the catalytic cleft of smooth muscle MLCK (22, 23). Moreover, hcTnI's NTE contains a series of positively charged amino acid residues upstream of the phosphorylatable serines 22/23, which are responsible for specific interactions with the catalytic core of protein kinases (24). Specifically, cTnI's NTE has an arginine in the P-3 position, and the analogous residue in RLC has been proposed to be crucial for binding to two conserved glutamate residues found in the catalytic core of smooth and skeletal cMLCK (25). Mutation of the P-3 arginine to alanine strongly reduced smooth and skeletal MLCK activity toward RLC (22). The sequence conservation with the catalytic core of cardiac MLCK therefore suggests a similar mechanism of substrate recognition of hcTnI's NTE by cMLCK (11).

Similar to cMLCK, another member of the same family of calmodulin-dependent kinases, protein kinase D (PKD), has been shown to monophosphorylate cTnI on serine 22 or 23 in response to α-adrenergic stimulation of cardiomyocytes (19). Moreover, PKD phosphorylates other sarcomere-associated proteins, such as cMyBP-C and telethonin, suggesting that cMLCK might also have broader substrate specificity. In fact, cMLCK efficiently phosphorylates the isolated tail domain of human telethonin (amino acids 104–161) on two sites *in vitro* (Fig. S5*A*). However, cMLCK did not phosphorylate N-terminal domains of cMyBP-C (C0C2), even at very high enzyme-to-substrate ratios (Fig. S5*B*), consistent with published results indicating that cMyBP-C is not a substrate for cMLCK (7).

The activity of kinases can also be controlled by tethering them to specific subcellular compartments, *e.g.* PKA activity is locally regulated within cells via interactions with so-called protein kinase A–anchoring proteins, which bind PKA with high specificity and affinity (26). In contrast, cMLCK has mainly been found diffusely localized in the cytosol of cardiomyocytes with some striated patterns (14, 27), suggesting that cMLCK can access myofibril and nonmyofibril compartments of cardiomyocytes. Interestingly, the striated patterns do not colocalize with myosin, its primary substrate, but with actin.

The upstream regulators for cMLCK's localization and substrate specificity remain unclear. Perhaps cMLCK's N-terminal extension, which includes putative phosphorylation sites for several physiologically and pathophysiologically relevant kinases (12), can regulate its localization, substrate specificity, and function. Consistent with this idea, adrenergic and prostanoid F receptor stimulation have been shown to increase RLC phosphorylation, probably by stimulation of cMLCK activity (11, 28).

### Functional implications of cTnI phosphorylation by cMLCK for cardiac myofilament function

The phosphorylatable N-terminal extensions of cRLC and cTnI are intrinsically disordered regions, which facilitates substrate recognition by protein kinases and allows control of downstream regulatory mechanisms via dynamic protein–protein interactions. Consistent with this idea, the NTEs of cTnI and cRLC have been shown to regulate myofilament function in a phosphorylation-dependent manner (7, 29).

Although serines 22 and 23 in the cTnI NTE are subject to phosphorylation by a series of protein kinases (*e.g.* PKA, PKD, and PKC), and bis-phosphorylation has been proposed to be necessary for the downstream functional effects (30), a recent study has shown that monophosphorylation on Ser^22^ or Ser^23^ is sufficient to reduce myofilament calcium sensitivity to the same extent as bis-phosphorylation (19). This suggests that monophosphorylation of cTnI on serine 23 by cMLCK is functionally significant for the myocardium and that different phosphorylation sites and combinations thereof might have different functional effects (31). Moreover, cTnI phosphorylation by PKA is hierarchical with serine 23 being phosphorylated before serine 22 (32, 33), further suggesting that cMLCK phosphorylation of serine 23 might prime serine 22 for phosphorylation by PKA and other kinases. Phosphorylation of hcTnI's NTE by cMLCK therefore represents a novel signaling mechanism in the regulation of cardiac contractile function under physiological and, potentially, pathophysiological conditions.

In the presence of human cardiac troponin, cMLCK incubation of ventricular trabeculae increased cross-bridge kinetics via phosphorylation of RLC. However, the associated increase in calcium sensitivity observed in native rat cardiac fibers is largely abolished (7), suggesting that phosphorylation of human cTnI in the same preparations mitigated the increase in calcium sensitivity associated with phosphorylation of RLC (Fig. 5). Moreover, cMLCK incubation reduced the cooperativity of myofilament calcium activation in the presence of human cTnI, as indexed by a decrease in the Hill coefficient *n_H_*, further supporting the hypothesis that cTnI phosphorylation by cMLCK modulates the functional effects of cRLC phosphorylation.

These results therefore suggest that Ca^2+^-dependent activation of cMLCK in the human heart has two functional consequences: activation of the thick filament structure via phosphorylation of cRLC, associated with increased calcium sensitivity, cross-bridge kinetics, and isometric force production (6, 7), and decreased calcium sensitivity of the thin filaments, associated with a faster rate of relaxation via phosphorylation of cTnI (29). The net result is increased force production and an increased rate of myofilament activation and relaxation without significantly altering the calcium sensitivity of the myocardium. This suggests that cMLCK can coordinate thick and thin filament activation states in the human heart and modulate its inotropy and lusitropy in response to changes in the intracellular calcium transient. Simultaneous phosphorylation of cRLC and cTnI by cMLCK might contribute to the positive force–frequency relation and the slow force response to stretch of the healthy myocardium (34–36), which increases maximal force and the rates of myofilament activation and relaxation. However, other signaling pathways likely also feed into these regulatory mechanisms, which might act synergistically or compensatory to cMLCK signaling.

The functional effects of cMLCK activation in the human heart likely depend on the phosphorylation background of other sarcomeric regulatory proteins. During low β-adrenergic stimulation, cMLCK can phosphorylate cRLC and cTnI. However, at high levels of β-adrenergic stimulation, the cTnI NTE is phosphorylated by PKA, and cMLCK activation leads to phosphorylation of primarily cRLC, which increases cross-bridge kinetics, calcium sensitivity, and the rate of myofilament activation. Therefore, depending on the phosphorylation background, β-adrenergic stimulation and cMLCK activation might have antagonistic effects on myofilament function.

Phosphorylation of cRLC by cMLCK has been the focus of considerable attention in clinical research over the last few years because decreased levels of RLC phosphorylation have been associated with heart disease and heart failure (37). In contrast, increased levels prevent onset of heart disease in animal models (18), suggesting that modulation of RLC phosphorylation by increasing cMLCK activity is a potential target for development of novel therapeutic agents for heart failure. Our results suggest that cMLCK's physiological function is likely to be more complex, and this should be taken into account during development. Cardiac troponin I dephosphorylation because of β-adrenergic receptor desensitization during heart failure development contributes to cardiac muscle dysfunction via impaired length-dependent activation, the cellular analog of the Frank–Starling law of the heart (38). Increasing cTnI phosphorylation in the failing heart by modulating cMLCK activity is therefore a potential new avenue for development of new heart failure interventions.

### Species-specific modulation of heart muscle function by cMLCK

Our results suggest that there are likely fundamental differences between the physiological functions of cMLCK in rodent and human heart muscle. Although human and rodent cTnI have ∼92% sequence identity, human cMLCK specifically phosphorylates only the human but not the rodent protein isoform *in situ* (Fig. 3), with only minor phosphorylation of rat cTnI after prolonged incubation *in vitro* (Fig. 1*C*). The catalytic domains of human and rat cMLCK exhibit very high sequence identity and similarity (>98%), but the current results cannot exclude the possibility that rat cMLCK can phosphorylate rat cTnI.

The P+1 position following serine 23 is an alanine in rodent but an asparagine in human cTnI, and it has been shown previously that deletion of the analogous asparagine in RLC greatly reduces MLCK's activity toward RLC (22). The comparison suggests that the additional alanine interferes with substrate recognition of rat cTnI by cMLCK, likely by increasing the distance between serine 23 and the posterior residues that anchor the cTnI NTE to a specific pocket close to the catalytic core. These results demonstrate that small protein sequence variations between species can have significant effects on myofilament regulation and their associated functional consequences. In agreement with this hypothesis, pharmacological inhibition of PKA and PKC did not affect the contractile performance of isolated human donor and failing heart muscle, although similar interventions had a significant effect on isolated rabbit myocardium (39, 40), suggesting species-specific effects of kinase signaling. Moreover, sequence variations in the N terminus of human and mouse cMyBP-C, with the latter exhibiting eight additional amino acids, have been shown to significantly modulate its interaction with myosin (41).

The concept of species-specific myofilament regulation has more fundamental implications for the study of heart failure, its etiology, and the development of pharmacological interventions using animal models. Loss-of-function mutations in cMLCK have been associated with dilated cardiomyopathy in humans (15, 16), whereas cMLCK ablation leads to a hypertrophic phenotype in mouse models (3), consistent with the view that the molecular functions of cMLCK differ between rodents and humans.

## Experimental procedures

### Protein production

The catalytic fragment of human cardiac myosin light chain kinase was prepared as described previously (10) based on complementary DNA AJ247087. The human cardiac troponin complex was kindly provided by Mitla Garcia-Maya (King's College London). Rat cardiac troponin C, I, and T were cloned from a rat heart complementary DNA library (BioChain Inc.) into a pET3a vector, codon-optimized for bacterial expression, and separately expressed in BL21(DE3)-RIPL cells (Agilent Technologies) according to the manufacturer's instructions. Rat cardiac TnC was purified as specified before (42). rcTnI was expressed at 37 °C in inclusion bodies and purified on a CM Sepharose ion exchange column using 6 mol/liter urea (buffer composition: 50 mmol/liter Tris-HCl (pH 8.0), 1 mmol/liter EDTA, and 5 mmol/liter DTT) and a linear gradient of 0–0.5 mol/liter NaCl. Rat cardiac troponin T was expressed in the soluble fraction and purified using a similar method. The troponin components were mixed at a 1:1:1 ratio in the presence of 6 mol/liter urea, and the urea concentration was reduced by serial dilution against a buffer containing 50 mmol/liter Tris-HCl (pH 8), 300 mmol/liter NaCl, 5 mmol/liter MgCl_2_, 1 mmol/liter CaCl_2_, and 1 mmol/liter DTT. The salt concentration was reduced to 200 mmol/liter followed by 100 mmol/liter in the last two steps of dialysis. The complex was purified by ion exchange chromatography on a Resource-Q column with a two-step linear gradient of 0–200 mmol/liter NaCl over 5 column volumes and 200–450 mmol/liter NaCl over 30 column volumes. The final concentrated product was characterized by HPLC, ESI-MS, and size-exclusion multi-angle light scattering (SEC-MALS) (Fig. S1). All measurements were consistent with a 1:1:1 complex of rat cardiac troponin T, I, and C. Troponin was dialyzed into a K_2_ EGTA buffer (25 mmol/liter imidazole, 78.4 mmol/liter potassium propionate (KPr), 6.8 mmol/liter MgCl_2_, 10 mmol/liter K_2_ EGTA, and 1 mmol/liter DTT (pH 7.1)), and aliquots were stored at −80 °C. Na_2_ creatine phosphate (Na_2_ CrP) and Na_2_ ATP were supplemented before troponin reconstitution in trabeculae (15 and 5.65 mmol/liter, respectively).

### HPLC, ESI-MS, and size-exclusion multi-angle light scattering

Analytical-scale HPLC was performed with an Agilent 1200 series system on octadecyl carbon chain (C18)–bonded silica columns (5-μm pore size, 4.6 × 250 mm, 218TP54, Vydac). The solvent system was HPLC-grade H_2_O (W/0106/PB17, Fisher Scientific) and HPLC-grade acetonitrile (ACN; A/0627/PB17, Fisher Scientific). Buffer A consisted of HPLC-grade H_2_O containing 0.1% (v/v) HPLC-grade TFA (T/3258/04, Fisher Scientific), and buffer B consisted of 80% (v/v) ACN in HPLC-grade H_2_O containing 0.1% (v/v) TFA.

Mass spectrometry analysis was performed on the Agilent Series 1100 LC-MS system in positive mode of ESI. The solvent system was as follows. Buffer A consisted of LC-MS grade H_2_O (W/0112/17, Fisher Scientific) containing 0.1% (v/v) formic acid (06440, Fluka), and buffer B consisted of 80% (v/v) ACN in MS-LC–grade H_2_O containing 0.1% (v/v) formic acid. Data analysis was performed with LC/MSD ChemStation software.

SEC-MALS was performed on a Superdex 200 5/150 GL column (GE Healthcare) in SEC-MALS buffer (50 mmol/liter Tris-HCl (pH 7.4), 5 mm MgCl_2_, 1 mmol/liter CaCl_2_, 100 mm NaCl, and 100 mmol/liter tris(2-carboxyethyl)phosphine). Recombinant troponins were extensively dialyzed against SEC-MALS buffer before experiments. Light scattering and refractive index were measured on a Mini DAWN and OPTILAB DSP (Wyatt Technology, UK), respectively. Data were analyzed with custom-written software.

### Preparation of cardiac myofibrils and ventricular trabeculae

All animals were treated in accordance with the guidelines approved by the United Kingdom Animal Scientific Procedures Act (1986) and European Union Directive 2010/63/EU. Wistar rats (male, 200–250 g) were sacrificed by cervical dislocation without anesthetics (schedule 1 procedure in accordance with the United Kingdom Animal Scientific Procedures Act, 1986), and demembranated right ventricular trabeculae were prepared as described previously (42). Some trabeculae were prepared in the presence of 10 μmol/liter H-89 and 1 μmol/liter staurosporine and dissolved in SDS-PAGE loading buffer for analysis of protein phosphorylation levels.

Human samples were obtained after informed consent and with approval of the institutional review board of the University of Kentucky (08-0338-F2L; approval date: January 18, 2017) and the King's College London Research Ethics Panel (LRS-16/17-4698; approval date: June 15, 2017). This investigation conformed with the principles of the Declaration of Helsinki (1997). Marmoset (*C. jacchus*) ventricular tissue was kindly provided by the Biological Service Unit of King's College London. Cardiac myofibrils were prepared as described previously (43). For dephosphorylation experiments, cardiac myofibrils were washed three times in λPP assay buffer (50 mmol/liter HEPES, 100 mmol/liter NaCl, 2 mmol/liter DTT, 1 mmol/liter MnCl_2_, 0.01% (v/v) Brij-35 (pH 7.5)). λPP was added to a final concentration of 4000 units/ml, and the mixture was incubated at 30 °C for 1 h. After incubation, CMFs were washed three times with relaxing buffer (20 mmol/liter MOPS (pH 7), 35 mmol/liter NaCl, 5 mmol/liter MgCl_2_, 1 mmol/liter EGTA, 1 mmol/liter CaCl_2_, 1 mmol/liter DTT, *p*Ca 9) without ATP to remove Mn^2+^ and λPP.

### In vitro and in situ kinase assays

Cardiac troponins were gel-filtered into cMLCK assay buffer (50 mmol/liter HEPES, 50 mmol/liter NaCl, 2 mmol/liter MgCl_2_, 1 mmol/liter CaCl_2_, and 1 mmol/liter DTT) via NAP5 columns (GE Healthcare). Concentrations were determined by UV absorbance at 280 nm using calculated extinction coefficients, and reactions were started by addition of calmodulin and cMLCK. Reactions were stopped by addition of SDS-PAGE loading buffer. Samples were denatured at 100 °C for 2 min, and phosphospecies were separated by Phos-tag^TM^ SDS-PAGE (containing 50 μmol/liter Phos-tag reagent and 100 μmol/l MnCl_2_). Gels were subsequently stained with Coomassie, and band intensities were quantified using ImageJ. CMFs (2–4 mg/ml) were phosphorylated *in situ* by incubation in cardiac activating solution (25 mmol/liter imidazole, 15 mmol/liter Na_2_ CrP, 58.7mmol/liter KPr, 5.65 mmol/liter Na_2_ ATP, 6.3 mmol/liter MgCl_2_, 10 mmol/liter CaCl_2_, 10 mmol/liter K_2_ EGTA, and 1 mmol/liter DTT (pH 7.1)) containing 25 μmol/liter blebbistatin and 1–2 μmol/liter cMLCK/CaM (0.5 nmol cMLCK/CaM per 1 mg of CMF protein) for 1 h at 30 °C. After incubation, CMFs were briefly washed in 10 mmol/liter Tris-HCl (pH 7) to remove EGTA. CMFs were dissolved in SDS-PAGE loading buffer, and samples were run on Phos-tag^TM^ SDS-PAGE as described above. The assays were repeated at least twice to make sure that they were reproducible and at least three times for quantitation.

### SDS-PAGE and Western blotting

After Phos-tag^TM^ SDS-PAGE, gels were washed for 10 min with transfer buffer (25 mmol/liter Tris-HCl (pH 8.3), 192 mmol/liter glycine, 0.08% (v/v) SDS, and 15% (v/v) methanol) containing 5 mmol/liter EDTA, followed by three washes in buffer without EDTA. Proteins were blotted onto nitrocellulose or PVDF membranes for 1–2 h at 1 mA/cm^2^ in transfer buffer using a Trans-Blot SD Semi-Dry Electrophoretic Transfer Cell (Bio-Rad). After blotting, membranes were blocked with TBS containing 0.05% (v/v) Tween 20 (TBS-T) and 5% (w/v) semiskimmed milk powder or 5% (w/v) BSA. Membranes were incubated with the following primary antibodies overnight in TBS-T containing 5% (w/v) semiskimmed milk powder or 5% (w/v) BSA: rabbit monoclonal anti-myosin light chain 2 (Abcam, 1:10000), mouse monoclonal anti-cardiac troponin I (MF4, GeneTex, 1:10,000), rabbit polyclonal anti-cardiac troponin I (Abcam, 1:1000), rabbit polyclonal anti-cardiac troponin I (pSer^22^ and pSer^23^, Cell Signaling, 1:1000), rabbit polyclonal anti-cardiac troponin T (Abcam, 1:1000), and rabbit monoclonal anti-pSer^282^ cMyBP-C (Enzo, 1:1000). Membranes were washed three times in TBS-T and incubated for 1 h at room temperature with secondary antibody (1:2000 dilution, HRP-conjugated donkey anti-rabbit IgG, GE Healthcare, NA934V, or HRP-conjugated goat anti-mouse IgG, Santa Cruz Biotechnology) in TBS-T containing 1% (w/v) semiskimmed milk powder or 1% (w/v) BSA. Blots were washed in TBS-T and immersed in ECL Plus reagent (GE Healthcare), and bands were visualized on a Bio-Rad Gel-Doc Imager. SDS-PAGE gels were stained with Pro-Q Diamond and SYPRO total protein stain according to the manufacturer's instructions (Life Technologies) and imaged on a Bio-Rad Gel-Doc Imager using the appropriate excitation and emission filter settings.

### Troponin exchange, λ protein phosphatase treatment, and experimental protocols for mechanical measurements of trabeculae

Endogenous troponin was exchanged by incubation of trabeculae in relaxing solution (25 mmol/liter imidazole, 15 mmol/liter Na_2_ CrP, 78.4 mmol/liter KPr, 5.65 mmol/liter Na_2_ ATP, 6.8 mmol/liter MgCl_2_, 10 mmol/liter K_2_EGTA, and 1 mmol/liter DTT (pH 7.1)) containing 1.5 mg/ml troponin complex and 30 mmol/liter 2,3-butanedione monoxime overnight at 4 °C. Trabeculae were mounted between a force transducer (Kronex, model A-801; resonance frequency, ∼2 kHz) and length controller (Aurora Scientific, model 312C). For dephosphorylation experiments, trabeculae were washed three times in MgCl_2_ rigor buffer (25 mmol/liter imidazole, 152.6 mmol/liter KPr, 1.5 mmol/liter MgCl_2_, 10 mmol/liter EGTA, and 1 mmol/liter DTT), followed by three washes in Mn^2+^ rigor buffer (25 mmol/liter imidazole, 152.6 mmol/liter KPr, 1.5 mmol/liter MgCl_2_, 1 mmol/liter MnCl_2_, and 1 mmol/liter DTT) and incubated for 1 h at room temperature in Mn^2+^ rigor buffer containing 3000 units/ml λPP. Subsequently, trabeculae were washed three times for 10 min in relaxing buffer, and sarcomere length was adjusted to ∼2.1 μm by laser diffraction.

Trabeculae were phosphorylated by incubation for 1 h at room temperature in activating solution (25 mmol/liter imidazole, 15 mmol/liter Na_2_ CrP, 58.7 mmol/liter KPr, 5.65 mmol/liter Na_2_ATP, 6.3 mmol/liter MgCl_2_, 10 mmol/liter CaCl_2_, 10 mmol/liter K_2_ EGTA, and 1 mmol/liter DTT (pH 7.1)) containing 30 mmol/liter BDM, 1 μmol/liter calmodulin, and 1 μmol/liter cMLCK. Subsequently, trabeculae were washed three times for 10 min in relaxing buffer, and sarcomere length was adjusted to ∼2.1 μm by laser diffraction.

Each activation was preceded by 2-min incubation in preactivating solution (25 mmol/liter imidazole, 15 mmol/liter Na_2_ CrP, 108.2 mmol/liter KPr, 5.65 mmol/liter Na_2_ ATP, 6.3 mmol/liter MgCl_2_, 0.2 mmol/liter K_2_ EGTA, and 1 mmol/liter DTT (pH 7.1)). Solutions with varying concentrations of free [Ca^2+^] were prepared by mixing relaxing and activating solutions using MAXCHELATOR software (RRID: SCR_000459). The dependence of force on free calcium concentration was fitted to data from individual trabeculae using nonlinear least-squares regression to the modified Hill equation:

(Eq. 1) $$\text{F}=\frac{1}{1+10^{n_{H}\left(p\text{Ca}-p{\text{Ca}}_{50}\right)}}$$

where *p*Ca = −log_10_ ([Ca^2+^]), *p*Ca_50_ is the negative logarithm of [Ca^2+^] corresponding to half-maximal change in F, and *n_H_* is the Hill coefficient. Trabeculae that showed a decline in maximal calcium activated force of more than 15% after the *p*Ca titrations were discarded.

The rate of force redevelopment was measured by a fast release and restretch protocol (44). Briefly, isometrically contracting trabeculae were released by 20% of their initial length (∼500-μs step response), held at the new length for ∼30 ms, and restretched to the original length. The time course of force redevelopment was fitted to a single exponential, yielding the rate constant *k*_tr_.

## Author contributions

I. R. S. and T. K. conceptualization; I. R. S. and T. K. data curation; I. R. S. and T. K. formal analysis; I. R. S., M. G., M. I., K. S. C., and T. K. writing-review and editing; B. B., S. P., M. G., M. I., K. S. C., Y.-B. S., and T. K. resources; B. B. and S. P. methodology; T. K. funding acquisition; T. K. writing-original draft.

## Supplementary Material

Supporting Information

## Footnotes

2 The abbreviations used are:

RLC: regulatory light chain
cMLCK: cardiac myosin light chain kinase
cTnI: cardiac isoform of troponin I
NTE: N-terminal extension
hcTnI: human cTnI
rcTnI: rat cTnI
cRLC: cardiac isoform of myosin regulatory light chain
ESI: electron spray ionization
CMF: cardiac myofibril
λPP: λ protein phosphatase
cMyBP-C: cardiac isoform of myosin-binding protein C
CaM: calmodulin
PKD: protein kinase D
SEC: size-exclusion chromatography
MALS: multi-angle light scattering
KPr: potassium propionate
CrP: Na_2_ creatine phosphate
ACN: acetonitrile.
