# Supplementary material for: Cardiac myosin regulatory light chain kinase modulates cardiac contractility by phosphorylating both myosin regulatory light chain and troponin I
Source: J Biol Chem. 2020 Feb 21;295(14):4398–410. doi: 10.1074/jbc.RA119.011945 (PMC7135997; doi:10.1074/jbc.RA119.011945)
Supplement: Supporting Information [file supp_295_14_4398__index.html]

Cardiac myosin regulatory light chain kinase modulates cardiac contractility by phosphorylating both myosin regulatory light chain and troponin I — cMLCK phosphorylates human cardiac troponin I — Cardiac myosin regulatory light chain kinase modulates cardiac contractility by phosphorylating both myosin regulatory light chain and troponin I — cMLCK phosphorylates human cardiac troponin I — Supporting Information 

# Cardiac myosin regulatory light chain kinase modulates cardiac contractility by phosphorylating both myosin regulatory light chain and troponin I

## Supporting Information

- Supporting Information (to be published online) - Supporting Information
