# Supplementary material for: Cardiac myosin regulatory light chain kinase modulates cardiac contractility by phosphorylating both myosin regulatory light chain and troponin I
Source: J Biol Chem. 2020 Feb 21;295(14):4398–410. doi: 10.1074/jbc.RA119.011945 (PMC7135997; doi:10.1074/jbc.RA119.011945)
Supplement: Supporting Information [file supp_RA119.011945_157186_2_supp_478816_q623tj.pdf]

Supplemental Information

Cardiac myosin regulatory light chain kinase modulates cardiac contractility by phosphorylating both myosin regulatory light chain and troponin I

Ivanka R. Sevrieva[1], Birgit Brandmeier[1], Saraswathi Ponnam[1], Mathias Gautel[1], Malcolm Irving[1], Kenneth S. Campbell[2], Yin-Biao Sun[1] and Thomas Kampourakis*[1]

[1]Randall Centre for Cell and Molecular Biophysics, and British Heart Foundation Centre of Research Excellence, King's College London, London, SE1 1UL, United Kingdom
[2]Department of Physiology, College of Medicine, University of Kentucky, Lexington, Kentucky, United States of America

Supplemental Information Figures and Figure Legends

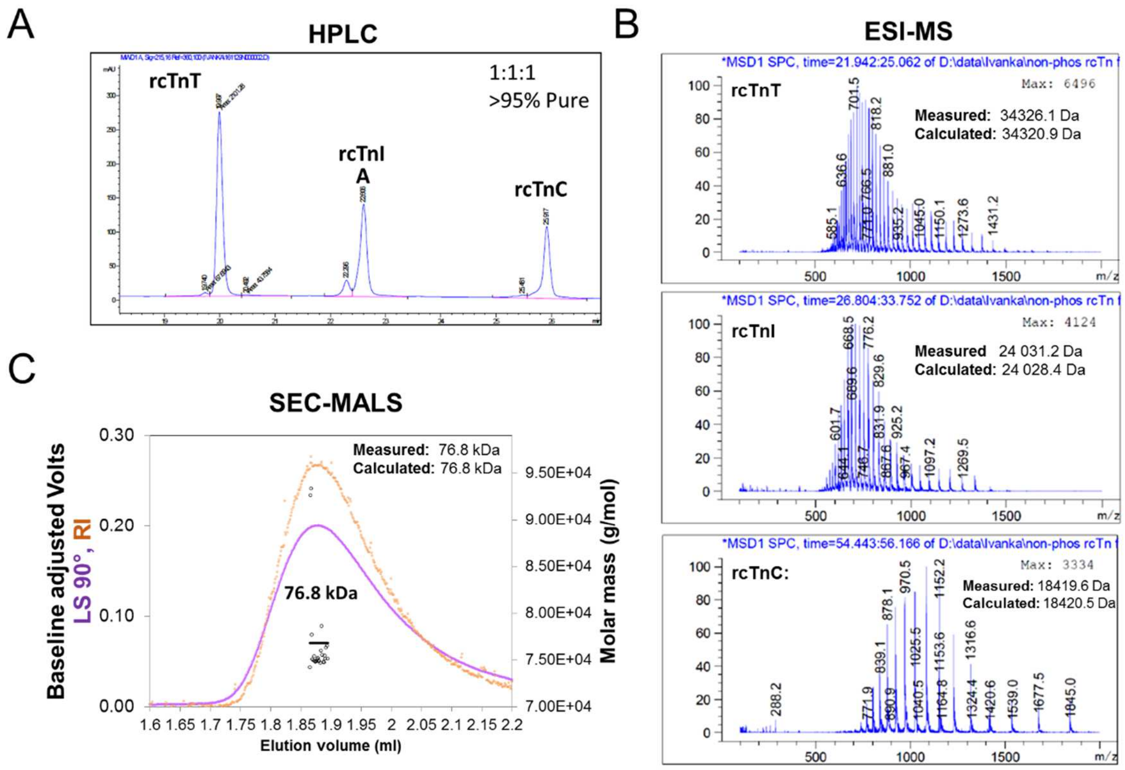

Figure S1. Analysis of recombinant rat cardiac troponin complex. (A) HPLC, (B) electron-spray ionization mass spectrometry and (C) SEC-MALS analysis of recombinant rat cardiac troponin complex (LS - light scattering, RI – refractive index).

Homo sapiens	ADGS-SDAARE----PRP-APAP-IRRRSS-NYRAYATEPH	old & new world monkeys
Pan paniscus	ADGS-SDAARE----PRP-APAP-IRRRSS-NYRAYATEPH	
Pan troglodytes	ADGS-SDAARE----PRP-APAP-IRRRSS-NYRAYATEPH	
Pongo abelii	ADES-NDAARE----PRP-APAP-VRRRSS-NYRAYATEPH	
Nomascus leucogenys	ADES-SDAARE----PRP-APAP-VRRRSS-NYRAYATEPH	
Cebus capucinus imitator	ADES-SDAAGE----PRP-APAP-VRRRSS-NYRAYAMEPH	
Rhinopithecus bieti	ADKS-SDVAGE----PRP-APAP-IRRRSS-NYRAYATEPH	
Rhinopithecus roxellana	ADKS-SDVAGE----PRP-APAP-IRRRSS-NYRAYATEPH	
Callithrix jacchus	ADES-SDAAGE----PRP-APAP-VRRRSS-NYRAYATEPH	
Saimiri boliviensis	ADES-SDAAGE----PRP-APAP-VRRRSS-NYRAYATEPH	
Aotus nancymaae	ADES-SDAAGE----PRP-APAP-IRRRSS-NYRAYATEPH	
Piliocolobus tephroscelus	ADKN-SDAAGE----PRP-APAP-IRRRSS-NYRAYATEPH	
Colobus angolensis palliatus	ADKN-SDAAGE----PRP-APAP-IRRRSS-NYRAYATEPH	
Theropithecus gelada	ADKS-SDAAGE----PRP-APAP-IRRRSS-NYRAYATEPH	
Macaca nemestrina	ADKS-SDAAGE----PRP-APAP-IRRRSS-NYRAYATEPH	
Macaca fascicularis	ADKS-SDAAGE----PRP-APAP-IRRRSS-NYRAYATEPH	
Macaca mulatta	ADKS-SDAAGE----PRP-APAP-IRRRSS-NYRAYATEPH	
Cercocebus atys	ADKS-SDAAGE----PRP-APAP-IRRRSS-NYRAYATEPH	
Mandrillus leucophaeus	ADKS-SDAAGE----PRP-APAP-IRRRSS-NYRAYATEPH	
Chlorocebus sabaeus	ADKS-SDAAGE----PRP-APAP-IRRRSS-NYRAYATEPH	
Papio anubis	ADKS-SDAAGE----PRP-APAP-IRRRSS-NYRAYATEPH	
Carlito syrichta	ADES-SHAAGD----PTP-APAP-VRRRSSANYRAYATEPH	Tarsier
Microcebus murinus	ADEN-GDLAGELPPAP-P--PAP-VRRRSSVNYRAYATEPH	Lemur
Propithecus coquereli	ADEN-GDVAGELPPAPAP-APAP-IRRRSSANYRAYATEPH	Lemur
Otolemu garnettii	ADEN-GDAAGELHQAPAP-APAP-VRRRSSANYRAYATEPH	Galago
Capra hircus	ADRSGSSTAGD----TVP-AP-P-VRRRSSANYRAYATEPH	
Bos taurus	ADRSGGSTAGD----TVP-AP-P-VRRRSSANYRAYATEPH	
Sus scrofa	ADRS-GDAAGS-----RP-APAP-V-RRSSANYRAYATEPH	
Equus ferus	ADQS-GNA-----------APPP-VRRRSSANYRAYATEPH	
Felis catus	AD-N-DDAAGC----PPP-APAP-VRRRSSANYRAYATEPH	
Canis lupus	ADES-GDAAGC----PPP-APAP-IRRQSSANYRAYATEPH	
Oryctolagus coniculus	ADES-RDAAGE----ARP-APAP-VRRRSSANYRAYATEPH	
Mus musculus	ADES-SDAAGE----PQP-APAP-VRRRSSANYRAYATEPH	
Rattus rattus	ADES-SDAAGE----PQP-APAP-VRRRSSANYRAYATEPH	
Coturni japonica	AEEE------E----PKP--P-P-LRRKSSANYRGYAVEPH	
Xenopus laevis	APEP-PKPA------PPPAAPPPLIRRRSSANYRSYATEPQ	

Figure S2. Multi-species sequence alignment of cardiac troponin I's N-terminal extension. Phosphorylatable serine residues corresponding to position 22/23 in the human sequences are highlighted in red.

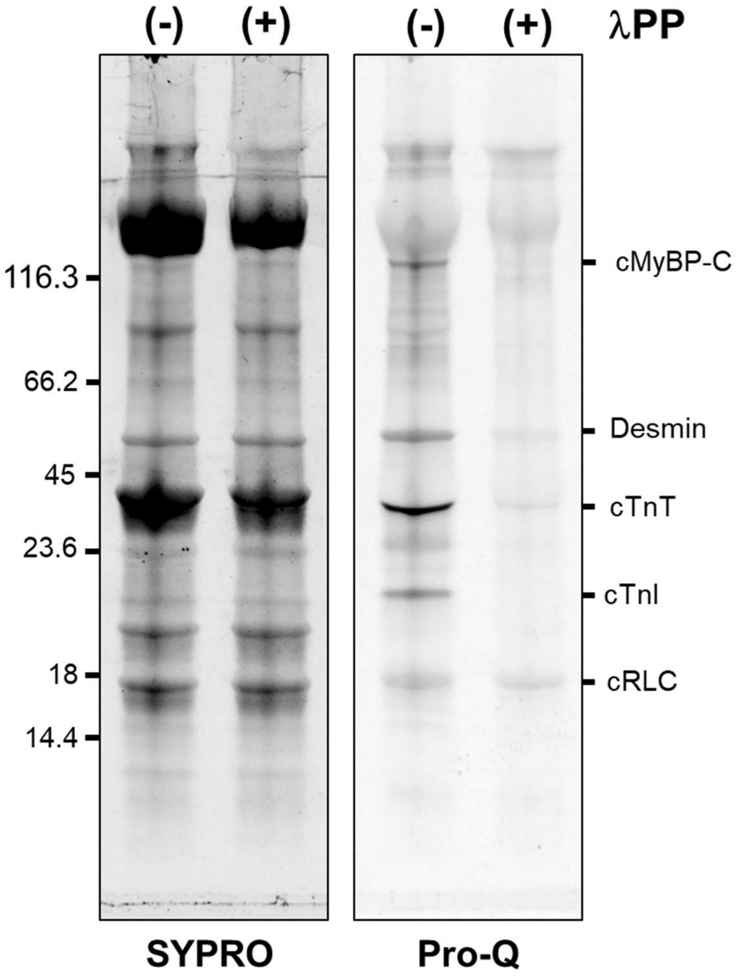

Figure S3. Total protein (left) and Pro-Q Diamond phospho-protein staining of rat cardiac myofibrils before (-) and after incubation (+) with λ-protein phospahatase (λPP). Key myofibrillar phospho-proteins are indicated on the right.

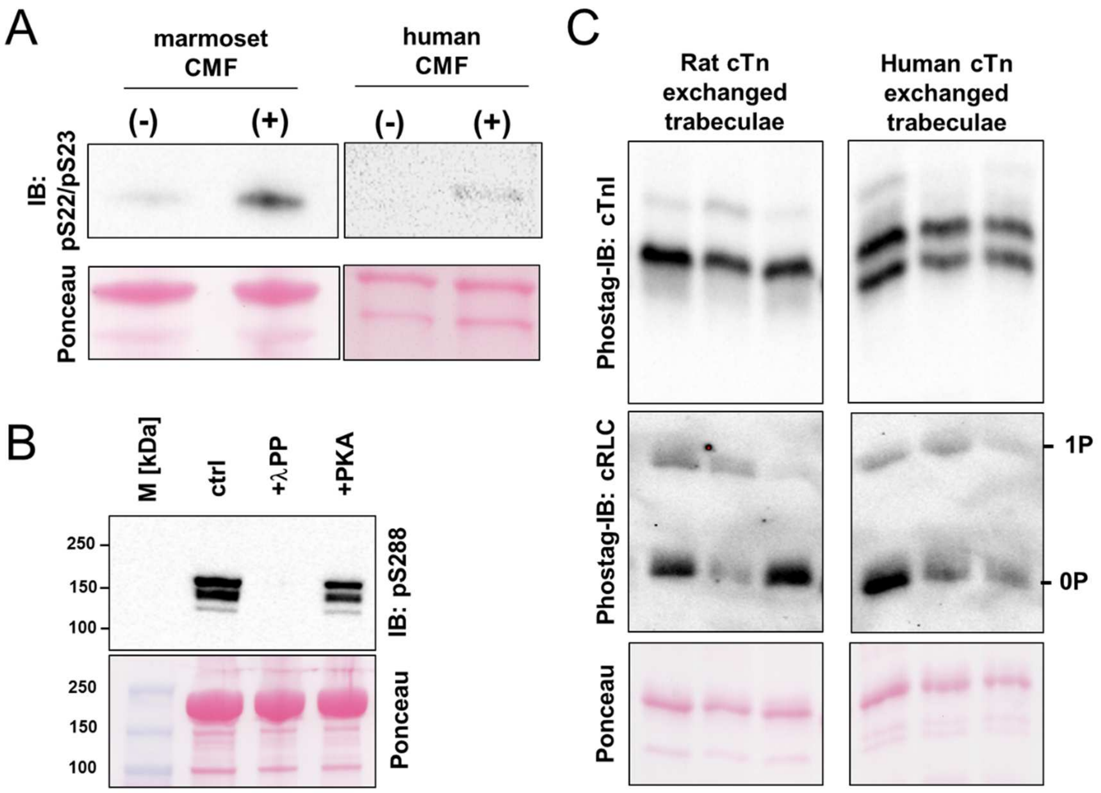

Figure S4. (A) Phosphorylation of cTnI before (-) and after cMLCK incubation (+) in λPP pre-treated marmoset and human CMFs confirmed by SDS-PAGE and Western-blot against serines 22/23-phosphorylated cTnI. Ponceau stains at the bottom show actin and troponin T bands as loading controls. (B) Dephosphorylation of cMyBP-C in rat CMF by λPP was confirmed by SDS-PAGE and Western-blot using a cMyBP-C phospho-specific antibody (pS288). As a control, PKA was added to dephosphorylated CMFs to back-phosphorylate cMyBP-C. Ponceau staining for myosin as loading control is shown at the bottom. (C) cTnI and cRLC phosphorylation level of experimental trabeculae after cMLCK treatment analysed by Phostag^{TM}-SDS-PAGE and Western-blot against cTnI (top) and cRLC (middle). Ponceau stains at the bottom show actin and troponin T bands as loading controls.

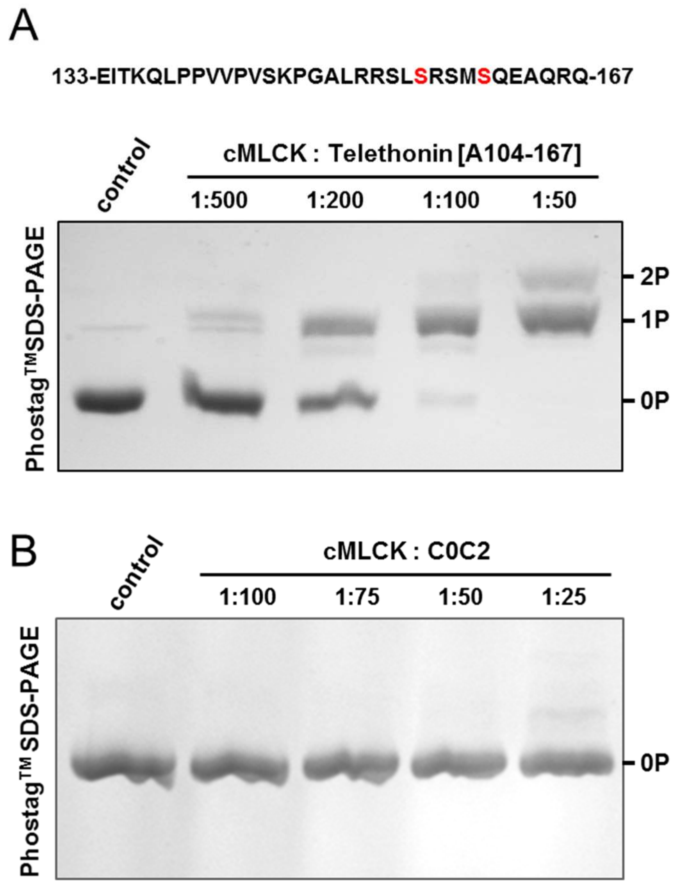

Figure S5. (A) Top: Protein primary sequence of human telethonin tail domain (amino acids 133 to 167). Phosphorylatable serine residues are highlighted in red. Bottom: *In vitro* kinase assay of cMLCK with the isolated telethonin tail domain at different enzyme-to-substrate ratios analysed by Phos-tag^{TM}-SDS-PAGE. Please note that cMLCK bis-phosphorylates telethonin. (B) *In vitro* kinase assays of cMLCK with cMyBP-C domains C0C2 at different enzyme-to-substrate ratios analysed by Phos-tag^{TM}-SDS-PAGE

Supplemental Information Tables and Table Legends

Table S1. Electron spray ionization mass spectrometry analysis of human cardiac troponin complex before (-cMLCK) and after cMLCK treatment (+cMLCK). Δm indicates the mass difference of troponin subunits before and after cMLCK treatment.

	- cMLCK		+ cMLCK	
	Calculated Mass [Da]	Measured Mass [Da]	Measured Mass [Da]	Δm [Da]
**hcTnT***	34459.07	34460.82	34459.95	-0.87
**hcTnI$**	24062.50	24063.61	24143.60	79.99
**hcTnC**	18370.45	18371.25	18370.93	-0.32

*human cTnT isoform 6; $additional glycine on N-terminus

Table S2. Summary of the effects of cMLCK treatment on mechanical properties of rat and human cardiac troponin exchanged trabeculae.

	+ Rat cTn		+ Human cTn	
	Before cMLCK	After cMLCK	Before cMLCK	After cMLCK
F_{min} [kPa]	1.23 ± 0.28	2.69 ± 0.75	1.13 ± 0.31	1.68 ± 0.33
F_{max} [kPa]	57.47 ± 7.58	59.32 ± 5.34	56.90 ± 7.56	58.08 ± 6.05
pCa_{50}	5.84 ± 0.01	5.91 ± 0.01***	5.93 ± 0.01	5.95 ± 0.01**
n_H	4.76 ± 0.24	4.44 ± 0.42	5.51 ± 0.19	4.11 ± 0.25*
$k_{tr\ 50\%max}$	7.32 ± 0.44	9.47 ± 0.48**	6.30 ± 0.66	8.25 ± 0.70**

Values indicate means ± SEM (n=4-5). Statistical significance of differences was assessed with two-tailed, unpaired Student’s t-test:*p<0.05, **p<0.01, ***p<0.001.
